# An overview of current prenatal genetic screening and diagnosis guidelines

**DOI:** 10.1002/pmf2.70016

**Published:** 2025-04-10

**Authors:** Carmen M. A. Santoli, Sarah K. Dotters‐Katz, Teresa N. Sparks, Jeffrey A. Kuller

**Affiliations:** ^1^ Maternal Fetal Medicine and Medical Genetics and Genomics Fellow, Department of Obstetrics, Gynecology, & Reproductive Sciences University of California San Francisco San Francisco California USA; ^2^ Division of Maternal‐Fetal Medicine, Department of Obstetrics & Gynecology Duke University School of Medicine Durham North Carolina USA; ^3^ Department of Obstetrics, Gynecology, & Reproductive Sciences University of California San Francisco San Francisco California USA

**Keywords:** carrier screening, cell free DNA, prenatal diagnosis, prenatal genetics

## Abstract

The landscape of prenatal genetics continues to evolve rapidly, with improvements in processing speed and technology. Clinicians are tasked with staying current with the latest recommendations for prenatal genetic screening and diagnosis in order to provide patient‐centered and evidence‐based care. We present a review of 15 societal guidelines that have been published or reaffirmed between 2016 and 2023 from the American College of Obstetricians and Gynecologists (ACOG), Society for Maternal Fetal Medicine (SMFM), American College of Medical Genetics and Genomics (ACMG), and International Society for Prenatal Diagnosis (ISPD). We provide a summary of the current guidance for carrier screening, cell‐free DNA (cfDNA) screening, and prenatal diagnostic testing, and also discuss key genetic principles. In brief, there are several approaches to prenatal carrier screening that range from a few select conditions (hemoglobinopathies, spinal muscular atrophy, cystic fibrosis) to several hundreds through expanded carrier screening panels. Both ACOG and ACMG support prenatal (ideally preconception) carrier screening, although have differing guidance about the optimal approach and number of conditions to screen. With regards to cfDNA screening, ACOG, SMFM, ACMG, and ISPD recommend offering all patients the option to screen for common aneuploidies, with consideration of sex chromosome aneuploidies after counseling. While ACOG, SMFM, and ISPD do not endorse microdeletion screening with cfDNA, ACMG supports cfDNA screening for 22q11.2 deletion syndrome. The societies are unanimous in recommending against cfDNA evaluation of rare autosomal trisomies. Finally, in terms of diagnostic testing, ACOG, SMFM, ACMG, and ISPD recommend offering chromosomal microarray for evaluation of fetal structural anomalies, stillbirth, and confirmation of screening results. Next‐generation sequencing with fetal exome or genome sequencing is recommended by ACMG, ISPD, and SMFM for evaluation of fetal anomalies following normal karyotype and/or microarray. ACOG, on the other hand, does not currently endorse fetal exome or genome sequencing outside of a research setting. Clinicians must have robust genetic literacy in order to understand current guidance and testing methodologies, discuss the benefits and limitations of genetic screening and diagnostic testing options, and engage in equitable and evidence‐based clinical practice.

## 1 INTRODUCTION

Rapid advancements in genetic technology and processing speed over the past decade have dramatically expanded the options available for prenatal genetic screening and diagnosis. Professional societies agree that prenatal genetic screening should be universally offered to all patients to evaluate for parental carrier status and common fetal aneuploidies [1]. Diagnostic testing with chorionic villous sampling (CVS) or amniocentesis can also be offered following a positive screening test, detection of fetal structural anomaly, or electively to provide definitive diagnostic information. It is well understood that information obtained through prenatal genetic screening and diagnostic testing can impact reproductive choice, antenatal care and delivery, postnatal preparedness, and future pregnancy planning. However, when individuals opt to pursue prenatal genetic screening or diagnostic testing, there are multiple considerations that inform the selection of which tests to pursue, such as family history, ultrasound findings, and personal preferences and values. With genetic technologies that continue to evolve rapidly, it is imperative for prenatal providers to stay current with the latest guidelines for best practices and understand available prenatal genetic screening and diagnostic tools.

We summarize and compare the latest 15 societal guidelines from the American College of Obstetricians and Gynecologists (ACOG), Society for Maternal Fetal Medicine (SMFM), American College of Medical Genetics and Genomics (ACMG), and International Society for Prenatal Diagnosis (ISPD) on carrier screening, cell‐free DNA (cfDNA) screening, and diagnostic genetic testing for prenatal diagnosis. We also review key genetic principles to inform our discussion of available tests and current recommendations.

### 1.1 Carrier screening

Carrier screening aims to identify asymptomatic individuals who carry one or more genetic variants associated with genetic disease, and to provide potentially actionable information about inheritance risk to guide pregnancy planning. Professional societies agree that carrier screening should be offered to preconception patients, or as early in pregnancy as possible if not previously completed. Preconception carrier screening allows time for result processing, partner testing, post‐test counseling, and, if positive results, making decisions about additional evaluations [2, 3]. Preconception individuals or reproductive partners who are identified as carriers of a genetic disease may choose to pursue artificial reproductive technology with preimplantation monogenic screening, donor gametes, adoption, or deferral of pregnancy. During pregnancy, prenatal diagnostic testing with CVS or amniocentesis can be considered to inform pregnancy management options or streamline postnatal care of an affected fetus [7].

There are three approaches to carrier screening: ethnic‐specific, pan‐ethnic, and expanded carrier screening (ECS). Historically, carrier screening was geared toward specific ethnic populations, such as individuals of Ashkenazi Jewish descent, known to be at increased risk for certain disorders such as Tay–Sachs disease. However, given the diversity in our multiracial society and that many patients lack a complete understanding of their ancestry, it has become increasingly challenging to use ethnic‐specific approaches. As a result, a pan‐ethnic approach was developed to screen all individuals regardless of reported ancestry for a select panel of genetic conditions. ECS is even more broad than pan‐ethnic screening in terms of the number of genetic diseases screened, and offers testing for up to hundreds of autosomal recessive and X‐linked conditions simultaneously. Although there is heterogeneity across ECS panels in the exact diseases that are tested, professional societies currently recommend that included diseases have a carrier frequency of at least 1/100–1/200 in any population, well‐defined phenotype, detrimental effect on quality of life with cognitive or physical impairment, early onset of disease features, available antenatal and/or postnatal interventions, and be identifiable with prenatal diagnosis [2, 3, 4, 5].

Careful pre‐ and post‐test counseling is necessary to cover the benefits and limitations of ECS, including the scope of diseases tested, downstream implications of identifying carrier status such as reproductive decision‐making and potential impacts to insurance coverage, possibility of health implications for the adults being screened, and residual risk. Importantly, carrier screening does not replace newborn screening (and vice versa), completely eliminate the risk of being a carrier for a heritable condition, or having a pregnancy affected by a genetic disease. The newborn screen is different in principle, as it assesses for the presence of a disease state rather than carrier status. Both newborn screening and carrier screening target actionable diseases during the childhood years such as phenylketonuria, although some ECS panels also evaluate for later‐onset conditions [3]. Importantly, X‐linked conditions may be identified with some ECS panels, and due to skewed X‐inactivation, female “carriers” may exhibit significant features of these diseases, such as with Fabry disease and Duchenne muscular dystrophy.

Both ACOG and ACMG provide guidelines regarding preconception and prenatal carrier screening [2, 3, 4, 5, 6]. ACOG committee opinion 690 (reaffirmed in 2020) and 691 (reaffirmed in 2020) acknowledge that all three carrier screening strategies as acceptable, depending on shared decision‐making based on the patient's family history and personal values [2, 4]. At present, each practice or health system may adapt their own routine for counseling about and ordering carrier screening. However, ACOG does recommend that all pregnant individuals or those considering pregnancy be offered at a minimum pan‐ethnic screening for spinal muscular atrophy with quantitative polymerase chain reaction; cystic fibrosis (CF) with targeted analysis for at least 23 known pathogenic variants; and hemoglobinopathies and thalassemias through a complete blood count and hemoglobin electrophoresis [2]. There are important clinical circumstances related to these conditions that require a more specialized approach to testing. First, in the setting of a family history of CF, targeted *CFTR* sequencing is indicated if the familial CF variant is known. Full sequencing of the *CFTR* gene is generally reserved for individuals with clinical evidence of CF who have not yet had confirmatory genetic testing, males with congenital bilateral absence of the vas deferens, or an infant with a positive newborn screen [2]. Second, hemoglobin electrophoresis is nondiagnostic in carriers of alpha thalassemia. Thus, when hemoglobin electrophoresis is normal and the complete blood count shows an unexplained low red blood cell mean corpuscular volume (<80 fL) with or without a suspicious family history, specific molecular genetic testing is indicated to detect alpha thalassemia carrier status.

ACOG further recommends that individuals with a family history of intellectual disability, autism spectrum disorder, Fragile X‐related disorder, or unexplained primary ovarian insufficiency or elevated follicular stimulating hormone before 40 years of age should be offered screening that includes Fragile X syndrome through molecular analysis of nucleotide expansion repeats. In addition, ACOG recommends that individuals with eastern and central European Jewish ancestry be additionally offered carrier screening that at a minimum includes Canavan disease, familial dysautonomia, and Tay–Sachs disease. ACOG discusses that screening for less common autosomal recessive conditions may also be considered for these populations, such as Bloom syndrome, familial hyperinsulinism, Fanconi anemia, Gaucher disease, glycogen storage disease type I, Joubert syndrome, maple syrup urine disease, mucolipidosis type IV, Niemann–Pick disease, and Usher syndrome [2]. ACOG recommends sequential carrier screening, meaning that if one partner is found to be a carrier, the other partner should then be offered screening [2, 4].

ACMG guidance differs from ACOG in several areas. In 2021, ACMG released a position statement which endorses that carrier screening should be ethnic and population neutral to improve equity among diverse and multiracial populations [3]. In addition, this statement supports concurrent partner testing, rather than sequential, when screening is performed during pregnancy in order to obtain results in a timely fashion.

ACMG categorizes carrier screening in an overlapping and tiered approach:

Tier 1: CF, SMA, and risk‐based screening
Tier 2: Tier 1 and conditions with ≥1/100 carrier frequency
Tier 3: Tier 2, conditions with ≥1/200 carrier frequency, and select X‐linked conditions
Tier 4: Tier 3 and conditions with <1/200 carrier frequency

ACMG recommends Tier 3 carrier screening, as Tiers 1 and 2 may miss opportunities to identify at risk couples who are carriers of diseases with lower population frequencies [3]. In a retrospective cohort study of patients who underwent ECS, altering the carrier threshold from >1/100 (Tier 2) to >1/200 (Tier 3) increased the detection of carriers by 14% and at‐risk couples by 4% [8]. This, however, assumes complete uptake of partner testing, which in reality is not always the case. Tier 3 includes carrier screening for 97 autosomal recessive conditions and select X‐linked conditions for the pregnant or preconception individual and autosomal recessive conditions for the male biological partner. While ACMG does not discuss an opt‐in approach to carrier testing for X‐linked conditions for pregnant or preconception individuals, some experts advise such an approach given growing recognition that female “carriers” of X‐linked diseases can have manifestations of those diseases themselves. ACMG recommends reserving Tier 4 for patients with suspected consanguinity or when personal or family medical history suggests that more extensive screening might be beneficial.

ACMG recommendations also differ from ACOG in terms of screening for CF variants. In 2001, ACMG recommended screening for 23 pathogenic CF variants in the US population [6]. This initial variant set was primarily derived from populations with non‐Hispanic White or Ashkenazi Jewish ancestry. Thus, the detection rate using this approach is lower in Black, Asian American, and Hispanic individuals as the specific variants tested account for a smaller portion of pathogenic CF variants in these populations [9]. Given that CF has been reported across nearly all ancestral backgrounds, ACMG revised their recommendations for CF screening in 2023 to include 100 variants that account for 95% of disease‐causing alleles in the US population [6]. At present, ACOG discusses the standard 23‐variant panel, although notes that panels with more CF variants may improve sensitivity for individuals of non‐White backgrounds [2].

It is important to counsel patients that a negative carrier screen for any particular genetic disease does not eliminate the risk of being a carrier or having an affected pregnancy. Carrier screening cannot identify all individuals at risk for a particular condition, as not all disease‐causing variants may be understood. It is likely that carrier screening using full gene sequencing will identify more at‐risk couples compared to targeted genotyping for select variants, such as with CF [10]; however, the clinical utility and cost‐effectiveness of these approaches require further investigation.

In summary, ACOG and ACMG agree on some principles of carrier screening, such as that it is best done preconception and diseases screened should meet several criteria including significant impacts to health at a young age. However, there are many differences between their recommendations, and ACMG advises a more expansive approach to ECS and CF carrier screening compared to ACOG. Given these differences and the wide breadth in available commercial carrier screening panels from which to choose, clinicians are tasked with developing their own standard approach that is consistently discussed with and offered to patients. Currently, ISPD does not have a position statement on carrier screening and SMFM is in the process of updating carrier screening guidelines.

### 1.2 Cell‐free DNA screening

Since its clinical implementation in 2011, noninvasive prenatal screening using cfDNA has progressively replaced maternal age‐based serum analyte screening for the common fetal aneuploidies (trisomy 21, 18 and 13). With technological advances shifting toward genome‐wide coverage, cfDNA has expanded with greater speed than supporting clinical studies to include sex chromosomes, sub‐chromosomal, and rare autosomal abnormalities. More than ever, clinicians are tasked with reconciling the marketing of such cfDNA platforms with evidence‐based clinical performance to guide test selection.

In brief, circulating cfDNA in pregnancy is a combination of maternal cfDNA derived predominantly from the hematopoietic system and fetal/placental cfDNA originating from cytotrophoblasts in chorionic placental villi [1, 11, 12]. Given this admixture of maternal and fetal/placental genetic information, there is a possibility of false‐positive and false‐negative information, particularly in situations such as confined placental mosaicism, vanishing twin, or maternal genetic abnormalities such as malignancy or mosaicism [13, 14]. Thus, cfDNA remains a screening test that requires diagnostic confirmation. The positive predictive value (PPV) and negative predictive value (NPV) of cfDNA depend on the population prevalence of disease and a priori risk of the disease [1, 15]. Comprehensive pretest counseling is imperative to discuss the performance, limitations, and possibility of incidental findings with cfDNA screening; similarly, post‐test counseling is essential to review clinical recommendations following a positive or inconclusive result and residual risk following a negative result [16]. Due to the possibility of both false‐positive and false‐negative results with cfDNA, CVS or amniocentesis are recommended for diagnostic certainty [1, 15, 17]. For chromosomes with high risk of confined placental mosaicism, such as rare autosomal trisomies (RATs), monosomy X, and trisomy 13, amniocentesis (rather than CVS) is advisable in the setting of a normal first trimester ultrasound [18]. Importantly, patients who desire definitive diagnostic testing should be offered chromosomal microarray (CMA) and additional tests as indicated [1, 17, 19].

#### 1.2.1 Aneuploidy

ACOG, SMFM, ACMG, and ISPD unanimously recommend that noninvasive prenatal genetic screening should be offered to all pregnant individuals for screening of the common aneuploidies including trisomy 21, 18, and 13 (Table 1) [1, 15, 17, 19]. Compared to serum analyte screening, cfDNA is more sensitive and specific for trisomy 21 (sensitivity 98.9%, specificity 99.9%), 18 (sensitivity 94.1%, specificity 99.9%), and 13 (sensitivity 100%, specificity 99.9%) [1]. Patients with high‐risk results should be offered prenatal diagnosis with karyotype to confirm aneuploidy and to distinguish between trisomy and translocation.

**TABLE 1:** Summary of societal recommendations regarding cell‐free DNA for prenatal genetic screening.

	ACOG [1] 2017 (reaffirmed 2020)	SMFM [17] 2015	ACMG [15] 2023	ISPD [19] 2023
Common aneuploidies	Recommended	Recommended	Recommended	Recommended
Sex chromosome aneuploidies	Offered after counseling	Offered after counseling	Recommended	Offered after counseling
Copy number variants (microdeletions or duplications)	Insufficient evidence	Insufficient evidence	Recommended for 22q11.2 deletion syndrome; insufficient evidence for genome‐wide CNVs or other select microdeletion/duplication syndromes	Insufficient evidence
Rare autosomal trisomies	—	—	Insufficient evidence	Insufficient evidence
Monogenic conditions	Insufficient evidence^a^	—	—	—

Abbreviations: ACMG, American College of Medical Genetics and Genomics; ACOG, American College of Obstetricians and Gynecologists; CNV, copy number variant; ISPD, International Society for Prenatal Diagnosis; SMFM, Society for Maternal Fetal Medicine.

^a^ ACOG Practice Advisory released in February 2019 (reaffirmed in 2024).

#### 1.2.2 Sex chromosome aneuploidy

Collectively, sex chromosome aneuploidies are the most common aneuploidies, with an incidence of 1 in 400 newborns [20]. Examples of sex chromosome aneuploidies include Turner syndrome (45, X), Klinefelter syndrome (47, XXY), Jacobs syndrome (47, XYY), and Triple X syndrome (47, XXX). Compared to the common aneuploidies, individual sex chromosome aneuploidy (SCAs) have lower prevalence, more variable expressivity, and may have more mild phenotypes. Although the sensitivity and specificity for SCAs is high, the PPV varies from 14.5% (95% confidence interval 7.0%–43.8%) for Turner syndrome to 97.7% (94% confidence interval 78.6%–100%) for Kleinfelter syndrome due to varying disease prevalence [21]. It is important to note that false positive results for Turner syndrome can result from mechanisms such as age‐related inactivation of the X chromosome or underlying maternal mosaicism [22]. The overall reported NPV for the four SCAs is reported to be 99.9%, although rare false‐negative results have been documented [21].

ACMG strongly recommends that cfDNA be offered to screen for SCAs in singleton gestations given its high sensitivity and specificity [15]. ACOG, SMFM, and ISPD do not specifically endorse cfDNA for SCA but state that it can be offered for individuals pursuing screening for the common aneuploidies after detailed counseling (Table 1) [1, 17, 19]. Specifically, patients should be counseled about the low PPV, variable phenotypes, and possibility of identifying underlying maternal abnormalities of the X chromosome [23]. Many content experts support an opt‐in approach for SCA screening using cfDNA to ensure that patients are informed about the risks and benefits of this testing.

#### 1.2.3 Microdeletions

Copy number variants (CNVs), such as microdeletions and ‐duplications, occur spontaneously or through familial inheritance [24]. Unlike the common aneuploidies, the incidence of CNVs does not vary with maternal age. Collectively, CNVs are common, affecting 1%–2% of pregnancies with a structurally normal fetus and 6% of pregnancies with a structural anomaly [25]. Some cfDNA platforms allow for screening of certain microdeletions such as 22q11.2 deletion syndrome, Prader–Willi syndrome, Angelman syndrome, 1p36 deletion syndrome, and Cri‐du‐chat syndrome. In addition, detection of genome‐wide CNVs may be available depending on the technology of the cfDNA screening test selected. Despite the high sensitivity and specificity reported, there remains concern about low PPV due to the low prevalence of individual microdeletion and ‐duplication syndromes [26]. Initial internal validation studies for detection of CNVs were based on admixtures of normal to abnormal DNA to represent typical fetal fraction and lacked complete data about confirmatory genetic testing [24]. More recently, a prospective study that is part of the SNP‐based Microdeletion and Aneuploidy RegisTry (SMART) evaluated 18,289 pregnant individuals who underwent single nucleotide–based cfDNA screening. Findings were a high sensitivity (83.3%) and specificity (99.9%) for 22q11.2 deletion syndrome, modest PPV of up to 52.6% when smaller deletions were tested in addition to the common 3Mb A‐D deletion, and a high NPV (99.9%) [27].

ACOG, ISPD, and SMFM do not recommend cfDNA screening for microdeletion or ‐duplication syndromes due to insufficient data regarding performance and clinical utility (Table 1) [1, 17, 19]. ACMG does recommend offering cfDNA screening for 22q11.2 deletion syndrome based on the SMART study noted above but maintains that there is insufficient evidence to screen for other CNVs using cfDNA [15]. It is important for clinicians to be aware that cfDNA panels may vary in being opt‐in versus opt‐out for CNVs depending on the laboratory. Patients who desire to obtain definitive information regarding fetal CNVs are recommended to pursue prenatal diagnosis with CMA. SMFM is currently updating its cfDNA guidance.

#### 1.2.4 Rare autosomal trisomies

Cell‐free DNA platforms that use genome sequencing (GS) can detect RATs, meaning trisomies other than those involving chromosomes 21, 18, and 13. The majority of RATs detected during pregnancy represent confined placental mosaicism or fetal mosaicism [15]. Due to limited clinical validation studies and unknown prevalence in the general population, ACOG, SMFM, ACMG, and ISPD do not recommend routine cfDNA screening for RATs (Table 1) [1, 15, 17, 19]. Importantly, if a RAT is identified, referral to genetic counseling is indicated and amniocentesis is advisable (rather than CVS) due to the high risk of confined placental mosaicism [18].

#### 1.2.5 Single‐gene disorders

Certain cfDNA platforms have started offering screening for single‐gene disorders of autosomal or X‐linked conditions such as Noonan syndrome, achondroplasia, and osteogenesis imperfecta. Although cfDNA for monogenic conditions are not yet addressed by SMFM, ACMG, or ISPD, ACOG issued a practice advisory in 2019 (reaffirmed in 2024) stating that there is insufficient evidence to recommend cfDNA for single‐gene disorders due to limited clinical validation studies and uncertain utility in screening the general population [28]. Patients desiring definitive information about the risk of a single‐gene disorder in their fetus should be offered genetic counseling and diagnostic testing as clinically appropriate.

### 1.3 Prenatal diagnostic testing

Definitive prenatal diagnosis has the potential to inform reproductive choice, antenatal care, postnatal preparedness, and future pregnancy planning. The option for prenatal diagnosis via CVS or amniocentesis should be offered to all pregnant individuals who desire definitive results during pregnancy in addition to those with fetal anomalies and abnormal prenatal genetic screening results. Transcervical or transabdominal CVS can obtain placental villi for genetic testing between 10‐ and 13 weeks’ gestation, whereas amniocentesis can be performed as early as 15 weeks’ gestation [29]. The risk of miscarriage is approximately 0.22% and 0.13%–0.27% for CVS and amniocentesis, respectively [29]. For patients undergoing CVS, it is important to discuss the approximately 1% chance of receiving karyotype or CMA results that reflect true fetal mosaicism or confined placental mosaicism [30]. If CVS is performed and mosaicism is identified, an amniocentesis is indicated to evaluate for mosaicism in the fetus along with genetics consultation [18]. There are several genetic tests available for further evaluation in this scenario, and the recommendation for which to pursue can be case dependent.

#### 1.3.1 Karyotype

**FIGURE 1:**
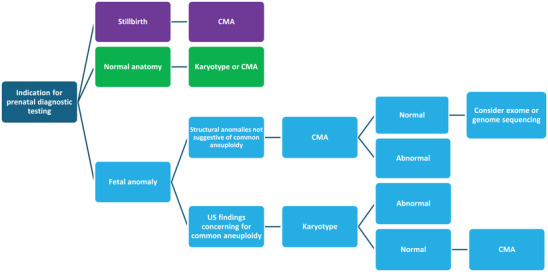
Prenatal diagnosis algorithm according to recommendations from Society for Maternal Fetal Medicine (SMFM), International Society for Prenatal Diagnosis (ISPD), and American College of Medical Genetics and Genomics (ACMG). CMA, chromosomal microarray. This algorithm is based on currently published societal guidelines. It is important to note that there may be differences in this algorithm based on more recently published evidence and clinical practice.

Karyotype is a chromosomal analysis that evaluates for autosomal and sex chromosome aneuploidies, large deletions and duplications (>5–10 Mb), balanced and unbalanced translocations, inversions, and other structural chromosomal abnormalities [29, 31]. Karyotype also can detect mosaicism at levels >10%–20% and polyploidy such as triploidy [29, 32, 33]. Accurate karyotypic analysis requires examination of chromosomes under the microscope by an experienced cytogeneticist. It requires cells to be cultured in the metaphase stage of mitosis, which can take 7–14 days. There is an increased risk of test failure with specimens from stillbirths since there are no viable cells to culture. Karyotype can be offered as a first test following cfDNA results suggesting a high risk of aneuploidy, if the fetal features are strongly suggestive of a particular aneuploidy. Karyotype may also be offered if patients prefer to avoid the chance of a variant of uncertain significance (VUS) with CMA (Figure 1) [29]. Karyotype is also indicated if there is a concern for an unbalanced translocation, in order to demonstrate the chromosomal locations of unbalanced material and inform recurrence risk counseling [31].

#### 1.3.2 Chromosomal microarray

CMA is a technique that can identify high resolution sub‐chromosomal deletions and duplications (as small as 50–100 kb), known as CNVs [29, 31, 34]. CMA can identify smaller deletions and duplications than detectable by karyotype, but it will miss balanced translocations and inversions [29, 31, 34]. CMA does not require dividing cells, thus can have a quicker turnaround time. A large multicenter trial through the National Institute of Child Health and Human Development demonstrated that CMA had a 6% incremental diagnostic yield over karyotype for fetuses with structural anomalies [35]. In addition, CMA identified clinically important CNVs in 1.7% of fetuses with normal ultrasounds and karyotypes. ACOG, SMFM, and ACMG recommend that CMA be offered to any patient undergoing invasive diagnostic testing, particularly those with fetal structural anomalies (Figure 1) [29, 31, 34]. CMA is preferred in cases of stillbirth, as it has a higher likelihood of yielding results without requiring dividing cells for culture [29, 31, 34].

Pre‐ and post‐test genetic counseling is important to discuss the possibility of obtaining a VUS and the variable expressivity of many genetic disorders. VUS are genetic changes occurring in 1%–3% of CMAs that cannot be categorized as benign or pathogenic due to limited data at present, although may be reclassified in the future. Parental samples may be useful to help interpret VUS results and identify if CNVs are inherited or de novo [31, 34]. While inheritance of a CNV from an unaffected biological parent may decrease the level of concern, it does not eliminate the possibility of disease manifestations. Patients must additionally be counseled on risk of discovering nonpaternity, consanguinity, and CNVs associated with adult‐onset disease [34].

#### 1.3.3 Next‐generation sequencing

More than half of fetuses with structural anomalies who have a normal karyotype also have normal CMA results [35, 36]. Next‐generation sequencing (NGS) provides nucleotide level information about single genes, and can identify inherited or de novo monogenic conditions that are not detected by karyotype or CMA. NGS is available through targeted gene panels, exome sequencing (ES) and GS [27]. Targeted gene panels evaluate a select subset of genes associated with a particular phenotype. Gene panels may be useful for cases where the fetal phenotype is suggestive of a specific disease category, such as a RASopathy or skeletal dysplasia. However, for fetal anomalies with a broad differential diagnosis and negative cytogenetic evaluation, ES or GS can be beneficial. ES examines the exons (protein‐coding regions) of the genome, which comprise only 1%–2% of the genome but are where the majority of human genetic disease is currently known to originate [37]. ES has been shown to increase the diagnostic yield by 31%–33% for fetuses with multisystem anomalies with normal karyotype and CMA [38, 39]. The yield of ES varies by phenotype, with a higher diagnostic yield for fetuses with hydrops and anomalies of the skeletal and cardiac systems and lower yield for pulmonary and abdominal anomalies [40]. Contributing details about abnormal phenotypes has been shown to improve the accuracy of variant detection with ES, highlighting the importance of detailed imaging and detection of abnormal features [41, 42]. GS examines both the exons and introns (noncoding regions) of the genome, evaluating approximately 98% of the total genome [37]. The incremental yield of GS over ES for fetal anomalies is an active area of clinical research, although preliminary evidence suggests a small incremental diagnostic yield but a faster turnaround time of prenatal GS over ES [43].

ES or GS can be pursued for the affected fetus alone or in combination with one or both biological parents—also referred to as duo or trio sequencing, respectively—to increase diagnostic yield by informing the inheritance and pathogenicity of detected variants. Broad sequencing with ES or GS often allows for greater identification of disease as this testing approach, unlike targeted panels, is not limited to a particular subset of genes [44]. ES and GS also allow for identification of genetic diagnoses not originally suspected due to limited fetal phenotypes detectable in utero or unique fetal manifestations of disease. Complete pretest consent for ES or GS includes the possibility of revealing nonpaternity or consanguinity, and the option for receiving secondary findings, which are genetic changes that increase risk of adult‐onset disease and have associated surveillance protocols [45]. It is also important to discuss the possibility of identifying an incidental finding, which is an unexpected diagnosis, such as a neurodevelopmental disorder, that may not be directly related to the fetal anomaly for which sequencing was initially pursued [46]. Finally, similar to CMA, it is also important to discuss the risk of obtaining a VUS, the possibility of variant reclassification over time with re‐analysis, and the possibility of impacted eligibility for supplemental insurance such as life insurance and disability insurance [47].

In a committee opinion on microarrays and NGS published in 2016 and reaffirmed in 2020, ACOG suggested that ES should be considered only in the context of clinical trials pending further peer reviewed validation studies on clinical utility [34]. Guidelines published by ACMG in 2020 and ISPD in 2022 (also endorsed by SMFM) recommended consideration of ES or GS for structural fetal anomalies following nondiagnostic karyotype and CMA [45, 48]. Overwhelming evidence has emerged over the last 5–10 years to support the increased diagnostic yield of ES and GS for pregnancies with fetal anomalies and nondiagnostic karyotype and/or CMA. Emerging evidence demonstrates support for the cost‐effectiveness and clinical utility of prenatal sequencing for fetal anomalies such as nonimmune hydrops fetalis, for example [49]. Prenatal ES or GS have the potential to provide families with a definitive diagnosis; identify genetic diseases that alter pregnancy decisions, clinical management, or eligibility for fetal therapies; and provide important context about recurrence risk in future pregnancies.

## 2 CONCLUSION

Clinicians should understand the benefits and limitations of prenatal genetic screening and diagnostic options to support their patients in selecting genetic tests. Counseling about prenatal genetic diagnostic options by or alongside genetics professionals is essential to inform pre‐ and post‐test counseling as well as selection of a testing strategy. Although ACOG, SMFM, ACMG, and ISPD converge on many recommendations for prenatal genetic screening and diagnosis, the societies offer varying opinions on certain topics such as cfDNA for 22q11.2 deletion syndrome, ECS, and the clinical application of fetal exome or GS. When interpreting the guidelines, it is important to consider the year in which they were published or affirmed, along with the peer‐reviewed evidence published in the interim, as this may explain some of the differences in recommendations. Consensus among obstetrical societies is needed to update and standardize recommendations with the latest information.

## CONFLICT OF INTEREST STATEMENT

The authors report no conflicts of interest.
